# The Effect of Lifestyle Intervention on Health-Related Quality of Life in Adults with Metabolic Syndrome: A Meta-Analysis

**DOI:** 10.3390/ijerph18030887

**Published:** 2021-01-20

**Authors:** Alba Marcos-Delgado, Natalia Hernández-Segura, Tania Fernández-Villa, Antonio J. Molina, Vicente Martín

**Affiliations:** 1 Departamento de Ciencias Biomédicas, Área de Medicina Preventiva y Salud Pública, Universidad de León, 24071 León, Spain; amarcd@unileon.es (A.M.-D.); nhers@unileon.es (N.H.-S.); ajmolt@unileon.es (A.J.M.); vmars@unileon.es (V.M.); 2 CIBER de Epidemiología y Salud Pública (CIBERESP), Instituto de Salud Carlos III, 28029 Madrid, Spain

**Keywords:** metabolic syndrome, quality of life, healthy lifestyle, health education, exercise, diet therapy

## Abstract

The aim of this meta-analysis was to assess the effects of a lifestyle intervention through health education on nutrition, physical activity, and healthy habits on physical and mental health-related quality of life (HRQoL), in adults with metabolic syndrome (MetS). The databases used were PubMed, WOS, and Scopus. The inclusion criteria were: observational, longitudinal and randomized clinical trial (RCT) study designs, adults (both sexes), with at least two criteria of MetS, lifestyle intervention and comparison with a control group, and a measurement of HRQoL with a validated questionnaire. We analyzed the Hedges’ g and SF-36 score. I^2^ statistics were calculated and possible publication and small study biases were assessed using Egger’s test and funnel plots. Seven RCTs were selected for meta-analysis, based on 637 study participants. Significant improvements were found in the physical dimensions of the HRQoL scores for subjects in the active intervention compared to the group that received general lifestyle information (Hedges’ *g* 0.61, 95% confidence interval (CI) = 0.31–0.91). Mental health-related quality of life was also significantly improved in the intervention group compared with the control group (Hedges’ *g* 0.84, 95% CI = 0.64–1.03). In conclusion, our results suggest that, according to the RCTs selected for this meta-analysis, a lifestyle intervention significantly improves HRQoL in all its domains.

## 1. Introduction

Cardiovascular diseases (CVD) are the leading causes of death in the world, accounting for a combined 15.2 million deaths in 2016. These diseases have remained the leading causes of death globally in the last 15 years [1]. Metabolic syndrome (MetS), a cluster of abdominal obesity, hyperglycemia, hypertriglyceridemia, low HDL (high density lipoprotein)-cholesterol, and hypertension, is known to be a strong risk factor for type 2 diabetes and is considered one of the most important preventable risk factors for CVD [2,3,4], whose prevalence is increasing. It is estimated that 39.9 ± 0.7% of the Asian population, 29.2 ± 0.7% of the European population, and 34.3 ± 0.8% of the adult US population suffers from MetS [5,6,7]. 

The main treatment for MetS prevention is a change in lifestyle through a multifactor approach based on education, regular physical exercise, and a healthy diet. An increasing number of studies support the idea that these changes in lifestyle were efficient in achieving the proposed goals for the treatment of MetS [6]. However, most physicians treat each component of MetS separately, prioritizing the treatment of those components that are easily amenable with drug treatment, given that it is easier to prescribe a drug to lower blood pressure, blood glucose, or triglycerides than to initiate a long-term strategy to change a person’s lifestyle [8].

It is important to highlight that MetS may lead to alterations in self-perceived well-being, since MetS has been linked to a decrease in health-related quality of life (HRQoL) [9,10]. Interestingly, these alterations in HRQoL may encourage the development of lifestyle changes more than the comorbidities associated with MetS itself [11]. Nevertheless, the relationship between MetS and HRQoL is complicated, with previous studies suggesting that the relationship may vary for the different components, physical and mental, of HRQoL. Moreover, some results are inconsistent, especially in relation to the mental component [12,13,14]. There are several validated questionnaires that measure the HRQoL, but the SF-36 (The Short Form-36 Health Survey) is the most widely used as an accurate way to measure self-perceived HRQoL. This questionnaire consists of 36 items that assess eight dimensions or scales, and these dimensions are grouped into two health components: the physical component summary (PCS) and the mental component summary (MCS). Each item received a numerical score that was encoded, summed up, and put on a scale from 0 to 100. The higher the score, the better the quality of life in the analyzed field [15,16,17]. 

In addition, there are also few studies evaluating the influence of lifestyle interventions on HRQoL in individuals with MetS [18,19,20,21,22,23,24,25,26,27,28]. There has been one systematic review of published studies which suggests that MetS is associated with reduced physical and mental HRQoL in cross-sectional studies, but, as of yet, no meta-analyses [29]. The systematic review of randomized clinical trials (RCTs) [29] reported improvements in metabolic parameters and HRQoL through lifestyle-based interventions. However, there was disagreement about which dimensions were most affected. 

Therefore, the aim of this meta-analysis is to assess the effects of lifestyle interventions on physical and mental HRQoL through health education on nutrition, physical activity, and healthy habits in adults with metabolic syndrome.

## 2. Materials and Methods 

### 2.1. Overview

This meta-analysis was reported in accordance with the Preferred Reporting Items for Systematic Review and Meta-Analysis (PRISMA) statement and registered in the prospective international register of systematic reviews PROSPERO with ID CRD42020176588.

### 2.2. Data Sources and Searches

**Figure 1:**
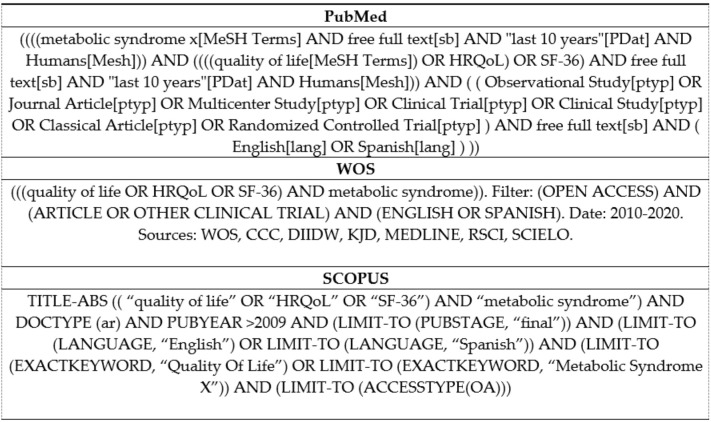
Search Strategy.

The search was conducted by the first author (A.M.D.) in March 2020. The search criteria for the three databases are detailed in Figure 1. The databases used were PubMed, WOS, and Scopus. Articles published in open access in the last 10 years and limited to English and Spanish language were selected. The terms: (metabolic syndrome x) AND ((quality of life OR HRQoL) OR SF-36)) were linked by logical operators. Observational, longitudinal, and clinical trials were included. 

All studies selected were approved by the relevant Ethics Committees, where the participants signed an informed consent form and complied with the provisions established by the Declaration of Helsinki. One author verified the ethical considerations in the included studies.

### 2.3. Study Selection

**Figure 2:**
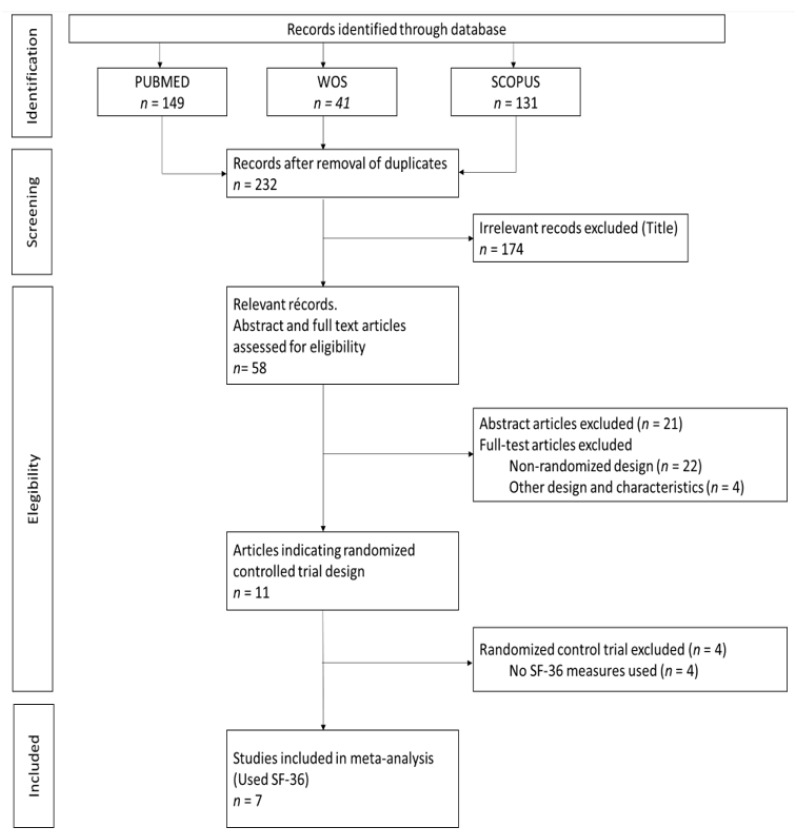
Preferred Reporting Items for Systematic Review and Meta-Analysis (PRISMA) Flowchart. WOS: Web of Science. SF-36: The Short Form-36 Health Survey.

The analysis of the search results and the selection of studies was carried out by two blinded and independent authors (A.M.D. and N.H.S.). First, a selection of the articles was made by title. Disagreements were resolved by consensus and the participation of a third senior author (T.F.V.) (Figure 2).

Once the selection was completed, one author (A.M.-D.) assessed the eligibility of the collected studies by verifying that they met the selection criteria set out under the acronym PICO (population, intervention, comparison, and outcomes). A second author independently verified the selection of studies.

The established inclusion criteria were: observational, longitudinal, and randomized clinical trial study design. The population was adults (≥18 years), both men and women, with at least two criteria of MetS. Lifestyle interventions included any intervention that focused on changes to diet, exercise, or motivational interviewing, or a combination of these in the intervention group. A control or usual care comparator group was required for comparison with the lifestyle intervention group. The measurement of HRQoL had to be conducted by means of a validated questionnaire. The quality measures of the randomized controlled trials were assessed using The Cochrane Collaboration’s risk assessment tool (Table 1). 

The established exclusion criteria were: children, adolescents, obesity only, other pathologies, and intervention with medicines.

### 2.4. Data Extraction

An Excel spreadsheet was created to extract the data, which was piloted in 3 studies by two independent and blinded reviewers (A.M.-D. and N.H.-S.). One author (A.M.-D.) independently performed the data extraction, a process verified by a second author (N.H.-S.), blinded to the results of the first author. The data collected were: reference and country, intervention(s), control treatment, study duration (weeks), percentage female (total), MetS criteria, mean age (total) years, and total sample.

### 2.5. Meta-Analysis

A random effects meta-analysis was undertaken to account for the differences in study design and location in the SF-36 score between the intervention group and the control group. 

For each outcome measure from SF-36, Hedges’ g and 95% confidence intervals (CIs) were calculated to assess the change in the experimental group compared to the control group. I^2^ statistics and 95% CIs were calculated to determine the degree of heterogeneity [30,31,32]. Possible publication and small study biases were assessed visually using funnel plots of the Hedges’ g against their standard errors, and then tested formally using Egger’s test [33]. All statistical analyses were performed using StataCorp 2019 (StataCorp LLC: College Station, TX, USA).

## 3. Results

### 3.1. Description of Studies

The electronic search identified 321 publications (Figure 2). We used three different databases that, when combined, found 89 duplicates, which were then excluded. The titles of the 232 remaining publications were reviewed, and 174 articles were found not to fulfill the inclusion criteria and were excluded. There were 58 relevant records, of which the abstract and full text were reviewed. Of these, 21 were excluded after reading the abstract because they did not fulfill the inclusion criteria. The full text articles reviewed included 22 non-randomized design studies and four that were of other designs and characteristics; these were excluded. Among the remaining 11 publications, four did not use the SF-36 questionnaire, two of which also had a high risk of bias (Table 1) [18,19,20,21,22,23,24,25,26,27,28]. Table 2 provides a detailed overview of the seven RCTs selected for meta-analysis, including information about the intervention(s), control treatment, study duration (weeks), percentage female, MetS criteria, mean age, and sample size. The earliest published study included was from 2015. Four of the studies were conducted in Asia (57.1%) [18,23,26,27], two in North America (28.6%) [19,22] and one in South America (14.3%) [24]. The study duration lasted from 12 to 36 weeks and the average participant was 54.7 years old. In addition, two studies were conducted on only male participants [22,23], while the remainder included both sexes. The total number of participants was 637, and all participants had at least two criteria for MetS. Interventions were described as lifestyle and exercise intervention, and control treatment was described as general information about nutrition and physical activity, or maintaining their current daily activities and exercise habits. For those studies with three comparison groups, their characteristics were evaluated to choose the intervention group to be included in the meta-analysis. In the case of Chiang et al. and Saboya et al. [23,24], the intervention group selected was the one that was subjected to an individualized and proactive intervention. For the Taylor et al. study, the two intervention groups were analyzed as one, according to the authors’ criteria [22]. All studies reported results for both the physical and mental health components of SF-36.

### 3.2. Study Quality and Risk of Bias

**Figure 3:**
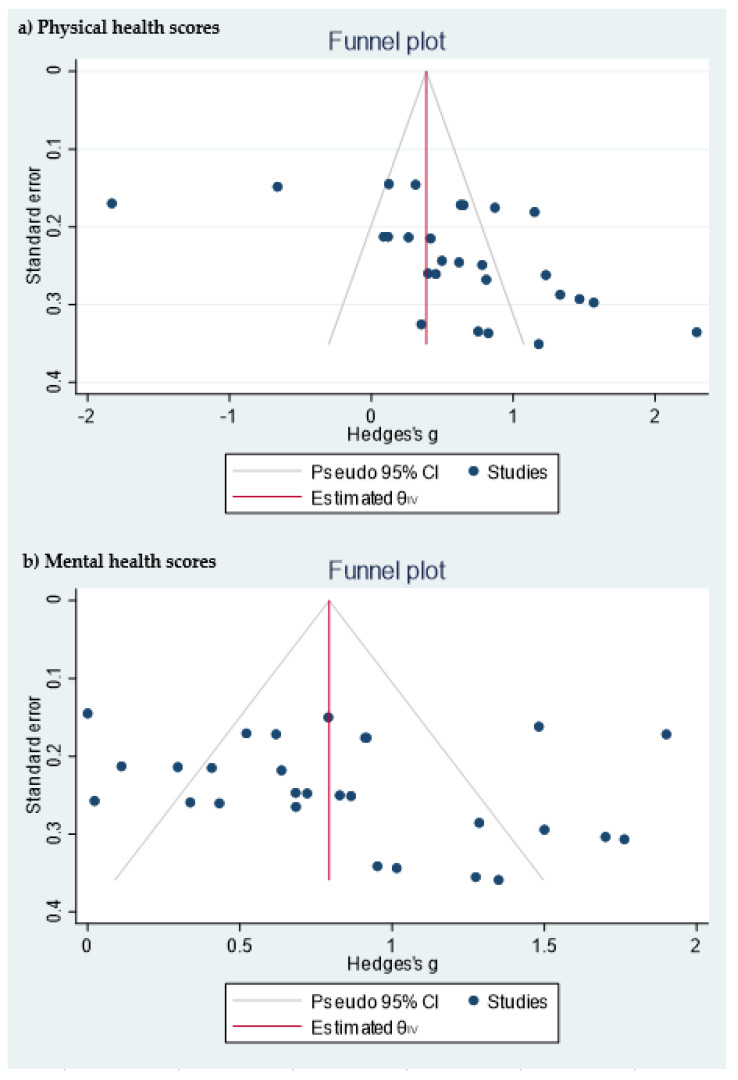
Funnel plot of the (**a**) physical health scores and (**b**) mental health scores.

Table 1 shows the quality measures of the randomized controlled trials. All studies included in the meta-analysis were at low risk of bias and reported using random sequence generation. Three of the studies had no information about allocation concealment, three provided this information, and one did not carry it out. Blinding was performed in three studies and none of the studies included reported incomplete and selective outcome data. In addition, publication bias was assessed using a funnel plot for physical and mental health scores (Figure 3). Egger’s test provided statistical evidence of funnel plot asymmetry in the physical health scores, suggesting the presence of a significant publication bias (*p* < 0.001). For the mental health scores, no significant publication bias was detected (*p* = 0.1078).

### 3.3. Physical Health-Related Quality of Life

**Figure 4:**
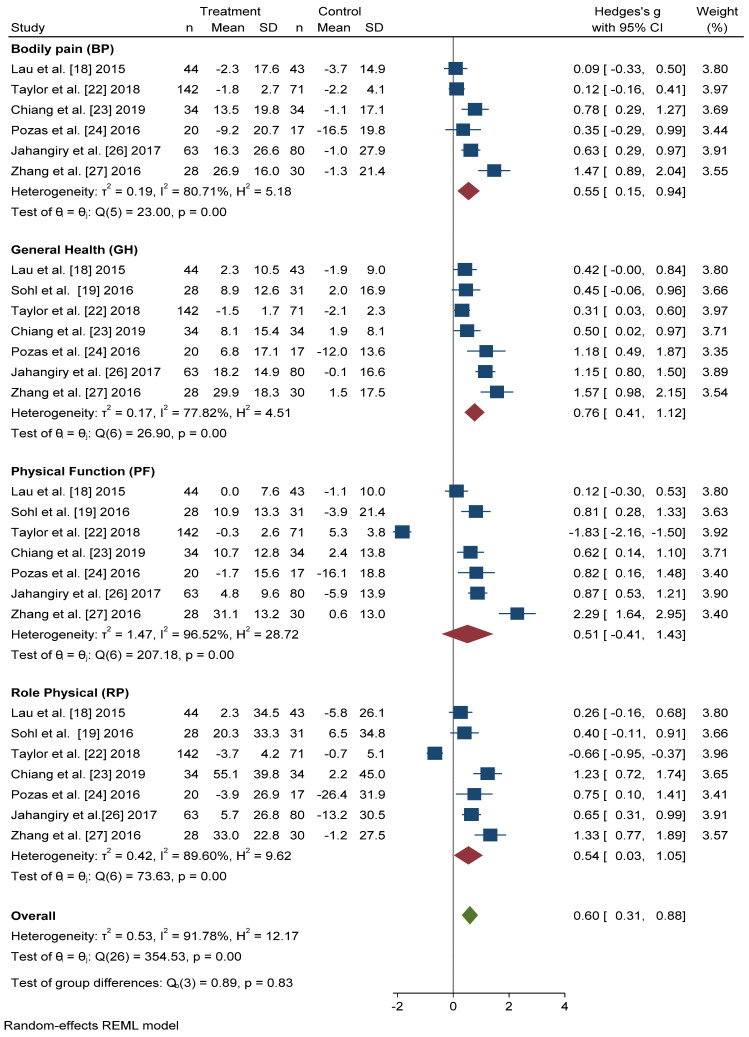
Forest plot of the physical health scores. Blue navy square centered at the point estimate of the effect size, with a horizontal line extending on either side of the square, representing the 95% confidence interval of the point stimate. Red diamond represents a confidence interval for each dimensions and green diamond represents a confidence a confidence interval for the overall effect size. REML: Restricted maximum-likelihood.

All seven studies that included the SF-36 questionnaire reported scores for the four physical dimensions in a format that permitted quantitative meta-analysis. In all dimensions, we found significant improvements in the intervention group with respect to the control group (Figure 4). The dimension with the greatest Hedges’ *g* difference between the two groups was General Health (GH): active intervention (*n* = 331) compared with the control group (*n* = 306) (Hedges’ *g* 0.76 points, 95% CI = 0.41–1.12, *p* < 0.001, I^2^ = 77.82%, 95% CI = 53.63–89.27).

The differences in Hedges’ *g* for Bodily Pain (BP) between the intervention group and the control group was 0.55 points, 95% CI = 0.15–0.94, I^2^ = 80.71%, 95% CI = 52.06–90.14. We also found significant improvements in the scores of Physical Function (PF) (Hedges’ *g* 0.51 points, 95% CI = −0.41–1.43, *p* < 0.001, I^2^ = 96.52%, 95% CI = 95.65–98.07) and Role Physical (RP) (Hedges’ *g* 0.54 points, 95% CI = −0.03–1.05, *p* < 0.001, I^2^ = 89.60%, 95% CI = 85.77–95.33) in the active intervention compared with the control group.

In overall change scores, significant improvement was found in subjects receiving the active intervention compared to the group that received general lifestyle information (Hedges’ *g* 0.61 points, 95% CI = 0.31–0.91). Substantial heterogeneity was present (I^2^ = 92.04%, 95% CI = 90.46–94.36). 

### 3.4. Mental Health-Related Quality of Life

**Figure 5:**
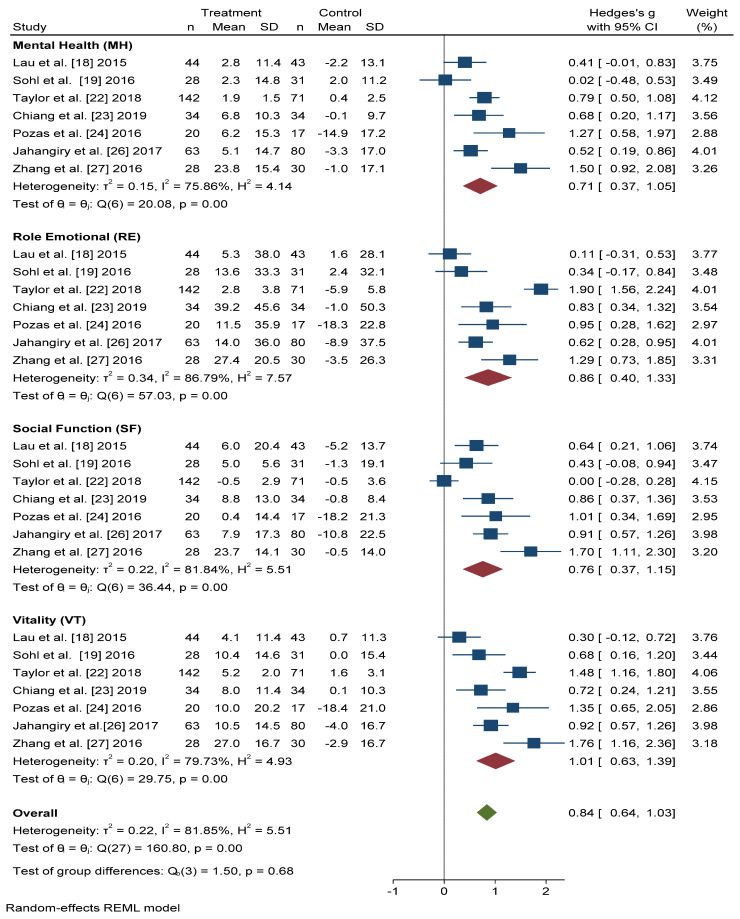
Forest plots of the mental health scores. Blue navy square centered at the point estimate of the effect size, with a horizontal line extending on either side of the square, representing the 95% confidence interval of the point stimate. Red diamond represents a confidence interval for each dimensions and green diamond represents a confidence a confidence interval for the overall effect size. REML: Restricted maximum-likelihood.

Figure 5 shows that the scores obtained in the Mental Health (MH) and Social Function (SF) dimensions were similar, and significant improvement was found with active intervention (*n* = 331) compared with the control group (*n* = 306) (Hedges’ *g* 0.71 points, 95% CI = 0.37–1.05, *p* < 0.001, I^2^ = 75.86%, 95% CI = 34.57–86.35 and Hedges’ *g* 0.76 points, 95% CI = 0.37–1.15, *p* < 0.001, I^2^ = 81.84%, 95% CI = 67.59–91.64, respectively).

For the Role Emotional (RE) domain, better results were also obtained in the intervention group than in the control group (Hedges’ *g* 0.86 points, 95% CI = 0.40–1.33, I^2^ = 86.79%, 95% CI = 80.84–94.22). The Vitality (VT) domain score improved, to a greater extent, in actively treated subjects compared with the control group (Hedges’ *g* 1.01, 95% CI = 0.63–1.39, I^2^ = 79.73%, 95% CI = 58.83–90.12).

In overall change scores, significant improvement was found in subjects receiving the active intervention compared to the group that received general lifestyle information (Hedges’ *g* 0.84 points, 95% CI = 0.64–1.03, I^2^ = 81.85%, 95% CI = 76.68–87.91). 

## 4. Discussion

In this meta-analysis of RCTs, we identified seven randomized trials that examined the impact of a lifestyle intervention on HRQoL in individuals with MetS. Significant improvements in HRQoL were found in those subjects who received the active lifestyle intervention compared with the usual care group. These improvements occur in all scores in the dimensions that make up the physical and mental component summary. 

There is a great deal of scientific evidence regarding the association between a healthy diet and physical activity in improving HRQoL in subjects with MetS [34,35], and now our study suggests that active lifestyle interventions in individuals with MetS were determinants in HRQoL. A previous systematic review was published in 2016 on studies examining the association between MetS and HRQoL [29], but to our knowledge, this is the first meta-analysis of the effect of lifestyle interventions on HRQoL in adults with MetS. Systematic review results show that MetS is significantly associated with worsening HRQoL and, furthermore, intervention studies for lifestyle modification in subjects with MetS demonstrate significant results in improving HRQoL after the intervention. However, some of these studies show association only in women, or only with depression or higher body mass index (BMI). 

Published scientific literature has reported that intensive lifestyle intervention improves anthropometric and metabolic parameters in individuals with MetS, and results in a significant association between overweight and obesity and impairment in the physical domain of HRQoL [36,37,38]. However, results of data on the impact of MetS on HRQoL are inconsistent. Given the importance of BMI in analyzing the relationship between HRQoL and MetS, it would be interesting to consider whether BMI would be a parameter to include in the measurement of the effectiveness of interventions. Only two of the RCTs included in this meta-analysis took into account the relationship between BMI and the HRQoL [22,24]. Usually, the physical spheres most affected were PF and GH, which could explain why a significant improvement was found in our study in the GH after the active intervention [13,39,40]. 

There was a significant improvement in all four domains of the mental health field, which was surprising, given that in previous studies the improvement in the mental spheres is confusing. In a cross-sectional study published by Vetter et al. [9], participants with MetS that were enrolled in a primary care-based weight reduction trial concluded that MetS was not associated with HRQoL as assessed using two generic instruments. However, a cross-sectional study published by Roohazfza et al. [41] in an Isfahan Cohort Study concluded the association of MetS with depression, anxiety, psychological distress, and quality of life. These differences may be due to the study design and the questionnaires used; our study shows that in the RCTs analyzed, after an intensive intervention in individuals with MetS, scores on the mental dimensions of the SF-36 questionnaire were better in the intervention group than in the control group.

This study had some limitations that need to be highlighted. First was the duration of the intervention, because the longest study was 36 weeks and did not show whether the improvement in quality of life was maintained over time. Second was the low number of RCTs published on these subjects and the small sample size included. We tried to overcome this limitation by performing the meta-analysis, which includes more than 600 individuals, and has therefore become one of our strengths. Third, the study of the differences between women and men is essential when discussing HRQoL and MetS. However, the characteristics of the studies did not allow the analyses to be carried out by sex. Furthermore, given the small sample size and the small number of selected articles, it would be possible to increase the variability. Finally, some analysis presents high heterogeneity, and due to the scarcity of articles it is not possible to unravel the differences between the different types of intervention and their duration. We believe that the results of the meta-analysis are promising, however, more and better research is needed in order to decrease the heterogeneity of the analyses.

An additional strength of this study was the inclusion of only published studies reporting SF-36 scores. SF-36 has been translated and validated in more than 50 countries and is the most commonly used measure. However, this was the first meta-analysis published on the impact of a lifestyle intervention on HRQoL in subjects with MetS, and it seems clear that there is a publication bias and significant heterogeneity in the evaluation of physical spheres. As such, the promising results detected in this meta-analysis are extremely important but they should be interpreted with caution. It is necessary to carry out further research in this field. 

## 5. Conclusions

In conclusion, our results suggest that, according to the RCTs selected for this meta-analysis, a lifestyle intervention significantly improves HRQoL in all its domains. Moreover, despite the importance of HRQoL as an outcome measure in medical research, the relationship between MetS and HRQoL is still poorly understood. Certainly, more RCTs are needed with a longer duration and larger samples. We recommend that all future trials of lifestyle interventions in individuals with MetS examine at least the influence on HRQoL of the improvement in anthropometric and metabolic parameters that make up MetS, and whether the improvement in HRQoL is sustained in the long term. 

## Figures and Tables

**Table 1:** Quality measures of the randomized controlled trials.

Source	Sequence Generation	Allocation Concealment	Blinding	Incomplete Outcome Data	Selective Outcome Reporting	Risk of Bias
Lau et al. 2015 [18]	No information	Yes	No	No	No	Low risk of bias
Sohl et al. 2016 [19]	Yes	Yes	Yes	No	No	Low risk of bias
Carvalho-Lima et al. 2017 [20]	No	No	No	Yes	Yes	High risk of bias
Sarwer et al. 2013 [21]	Yes	No information	No information	No	No	Low risk of bias
Taylor et al. 2018 [22]	Yes	No information	No	No	No	Low risk of bias
Chiang et al. 2019 [23]	Yes	Yes	Yes	No	No	Low risk of bias
Saboya et al. 2016 [24]	Yes	Yes	No information	No	No	Low risk of bias
Prasanth et al. 2018 [25]	Yes	No information	No information	Yes	Yes	High risk of bias
Jahangiry et al. 2017 [26]	Yes	No information	No information	No	No	Low risk of bias
Zhang et al. 2016 [27]	Yes	No	No	No	No	Low risk of bias
Fanning et al. 2018 [28]	Yes	Yes	Yes	No	Yes	Low risk of bias

**Table 2:** Summary of 7 randomized controlled trials included in meta-analysis.

Reference and Country	Intervention(s)	Control Treatment	Study Duration (Weeks)	% Female (Total)	MetS Criteria	Mean Age (Total), Years	Sample Size
Lau et al. [18] 2015 (China)	Yoga training consisting of 12 once-weekly, 60-min sessions (*n* = 44)	Maintain their routine activities and not begin any exercise (*n* = 43)	12	63	NCEP-ATP III	52.0 (7.46)	87
Sohl et al. [19] 2016 (USA)	Yoga and education (*n* = 26)	Education only (*n* = 33)	12	51	Standard MetS Criteria	58.0 (10)	59
Taylor et al. [22] 2018 (USA)	Supervised facility-based exercise intervention (*n* = 73) Home-based exercise intervention (*n* = 69)	Control group: Maintain their current daily activities and exercise habits (*n* = 71)	24	100	Standard MetS Criteria	58.3	213
Chiang et al. [23] 2019 (Taiwan)	Intervention group (IG) (*n* = 34) participants were given individually tailored, 12-week, telephone-based motivational counseling for modifying lifestyles. The other group (*n* = 32) received an educational brochure about lifestyle modification and coping with stress.	Control group only underwent routine outpatient clinical follow-up (*n* = 34)	12	100	NCEP-ATP III	IG: 63.1 (8.5) CG: 63.8 (7.3)	Total: 115 Groups included in the meta-analysis (CG and IG): 68
Saboya et al. [24] 2016 (Brazil)	Individual Intervention group (IG) (*n* = 28) participated in weekly individual appointments with psychology and nutrition teams and exercised regularly and the other group (*n* = 25) worked the change in lifestyle through the discussion of pre-defined themes of health education	Control group was the non-pharmacological intervention recommended by the main guidelines for the clinical management of MetS (*n* = 19)	36	55.5	Standard MetS Criteria	CG: 52.1 (7.2) IG: 51.6 (5.6)	Total: 72 Groups included in the meta-analysis (CG and IG): 37
Jahangiry et al. [26] 2017 (Iran)	Interactive lifestyle intervention with Healthy Heart Profile on nutrition, and physical activity (*n* = 63)	Sending e-mails every 3 weeks to visit the study website and read general information on nutrition and physical activity (*n* = 80)	24	33.7	NCEP-ATP III	44.2 (10.0)	143
Zhang et al. [27] 2016 (China)	Patient-centered cognitive behavioral therapy (PC-CBT) lifestyle intervention	Control group received a letter explaining basic lifestyle advice and general information about MetS risk factors (*n* = 30)	12	56.9	IDF 2005	48.6 (5.8)	58

MetS: Metabolic Syndrome. NCEP-ATP III: National Cholesterol Education Program Adult Treatment Panel III. IDF: International Diabetes Foundation. Standard MetS Criteria: elevated waist circumference (men greater than 102 cm; women greater than 88 cm), impaired fasting glucose (100–125mg/dL), elevated blood pressure (systolic ≥ 130 and/or diastolic ≥ 85), or diagnosis of hypertension and dyslipidemia (triglycerides ≥ 150 and/or HDL ≤ 40 for men; 50 for women). CG: Control group. IG: Intervention group.
